# Nitric oxide inhibits endothelial cell apoptosis by inhibiting cysteine‐dependent SOD1 monomerization

**DOI:** 10.1002/2211-5463.13362

**Published:** 2022-01-11

**Authors:** Hanlin Peng, Shangyue Zhang, Zaifeng Zhang, Xiuli Wang, Xiaoyu Tian, Lulu Zhang, Junbao Du, Yaqian Huang, Hongfang Jin

**Affiliations:** ^1^ Department of Pediatrics Peking University First Hospital Beijing China; ^2^ Key Laboratory of Molecular Cardiology Ministry of Education Beijing China

**Keywords:** cysteine, endothelial cell apoptosis, monomerization, nitric oxide, SOD1

## Abstract

Endothelial cell apoptosis is an important pathophysiology in many cardiovascular diseases. The gasotransmitter nitric oxide (NO) is known to regulate cell survival and apoptosis. However, the mechanism underlying the effect of NO remains unclear. In this research, by targeting cytosolic copper/zinc superoxide dismutase (SOD1) monomerization, we aimed to explore how NO inhibited endothelial cell apoptosis. We showed that treatment with the NO synthase (NOS) inhibitor nomega‐nitro‐l‐arginine methyl ester hydrochloride (L‐NAME) significantly decreased the endogenous NO content of endothelial cells, facilitated the formation of SOD1 monomers, inhibited dismutase activity, and promoted reactive oxygen species (ROS) accumulation in human umbilical vein endothelial cells (HUVECs); by contrast, supplementation with the NO donor sodium nitroprusside (SNP) upregulated NO content, prevented the formation of SOD1 monomers, enhanced dismutase activity, and reduced ROS accumulation in L‐NAME‐treated HUVECs. Mechanistically, tris(2‐carboxyethyl) phosphine hydrochloride (TCEP), a specific reducer of cysteine thiol, increased SOD1 monomer formation, thus preventing the NO‐induced increase in dismutase activity and the decrease in ROS. Furthermore, SNP inhibited HUVEC apoptosis caused by the decrease in endogenous NO, whereas TCEP abolished this protective effect of SNP. In summary, our data reveal that NO protects endothelial cells against apoptosis by inhibiting cysteine‐dependent SOD1 monomerization to enhance SOD1 activity and inhibit oxidative stress.

## Graphical abstract

Nitric oxide (NO) inhibits cysteine‐dependent copper/zinc superoxide dismutase (SOD1) monomerization to block the inactivation of SOD1 activity, thus protecting endothelial cells against oxidative stress‐induced apoptosis. The present study presents a possible new therapeutic principle behind NO in the treatment of oxidative stress‐related diseases including cardiovascular disease, aging, and cancer, and may facilitate the design and the clinical application of NO‐related drugs.

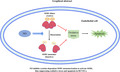

**Abbreviations**
DMEM/F12: Dulbecco’s modified Eagle’s medium/nutrient mixture F12
eNOS: endothelial nitric oxide synthase
HUVECs: human umbilical vein endothelial cells
L‐NAME: nomega‐nitro‐l‐arginine methyl ester hydrochloride
NO: nitric oxide
ROS: reactive oxygen species
SNP: sodium nitroprusside
SOD1: copper/zinc superoxide dismutase
TCEP: Tris(2‐carboxyethyl) phosphine hydrochloride
TUNEL: terminal deoxynucleotidyl transferase‐mediated dUTP nick‐end labeling

Endothelial cell apoptosis triggers vascular endothelial injury [1] and is the pathophysiological basis of numerous cardiovascular diseases, including atherosclerosis, hypertension, aneurysm, and pulmonary hypertension [2, 3, 4, 5, 6]. In endothelial cells, nitric oxide (NO) predominantly synthesized by endothelial nitric oxide synthase (eNOS) has an anti‐apoptotic effect, and therefore, it is a crucial vascular protective gasotransmitter [7]. The regulatory effect of NO on cell apoptosis involves the activation of guanylyl cyclase signaling, a decrease in intracellular Ca^2+^ levels, and a decrease in mitochondrial cytochrome c release [8, 9]. However, the molecular mechanism by which NO inhibits endothelial cell apoptosis has not been fully elucidated.

As one of the common mechanisms of endothelial dysfunction, the increase of reactive oxygen species (ROS) level is related to the occurrence of endothelial cell apoptosis [10]. Copper/zinc superoxide dismutase (SOD1) is the main antioxidant enzyme, which can scavenge superoxide anions. The upregulation of SOD1 activity leads to the elimination of superoxide anions, thereby increasing the resistance of endothelial cells to pro‐apoptotic stimuli, such as tumor necrosis factor α and oxidative damage, suggesting that SOD1 activation is a significant defense mechanism of endothelial cells [10, 11]. Previous studies have shown that NO levels are decreased in elderly hypertensive patients, accompanied by reduced SOD1 activity [12]. The left ventricle of spontaneously hypertensive rats treated with an alcohol‐free red wine extract exhibited increased NOS and SOD1 activities [13]. Treatment with the NO inhibitor nomega‐nitro‐l‐arginine methyl ester hydrochloride (L‐NAME) reduced the activity of SOD1 in the lungs of rats with acute lung injury and hyperbaric oxygen preconditioning [14]. These findings led us to hypothesize that NO might regulate SOD1 activity, which might be vital of the anti‐apoptotic effect of NO. While the mechanism by which NO regulates SOD1 activity is unclear.

The formation of SOD1 monomer leads to a decrease in its activity and is an early step in SOD1 aggregation, which causes familial amyotrophic lateral sclerosis (fALS) [15, 16]. NO can promote the α‐ and β‐subunit of soluble guanylyl cyclase monomer to form heterodimer to activate it [17]. However, whether NO acts on SOD1 monomers remains unclear. Thus, we examined possible function of NO on SOD1 monomerization to clarify the mechanism for SOD1 activation on endothelial cell apoptosis in our study.

## Materials and methods

### Western blotting

The level of SOD1 dimer and monomer in human umbilical vein endothelial cells (HUVECs) was detected as follows. The treated cells and the control cells were lysed in buffer (pH 7.5, 20 mm Tris, 1 mm EDTA with 0.5% Triton X‐100). After centrifugation, the supernatants were mixed with loading buffer (B1033, Applygen, Beijing, China), which contains 0.2% SDS without any reducing agent, incubated at room temperature, and loaded onto a 12.5% SDS/PAGE gel for protein electrophoretic separation. Before transfer onto nitrocellulose membrane (Amersham, USA), the protein was subjected to in‐gel reduction by incubating with transfer buffer including 2% β‐mercaptoethanol for 10 min [18].

The membrane was probed with primary antibodies against SOD1 dimer and monomer (1 : 1000; Cat No. ADI‐SOD‐100‐D; Enzo, Farmingdale, New York, USA) and β‐actin (1 : 3000; ZSGB‐Bio, Beijing, China). Then, the membrane was incubated with horseradish peroxidase‐conjugated corresponding secondary antibodies. The FluorChem M Multifluor System (ProteinSimple, San Francisco, California, USA) was used to visualize protein bands.

### Cell culture and treatment

Dulbecco’s modified Eagle’s medium/nutrient mixture F12 (DMEM/F12), which contains 10% fetal bovine serum (FBS), 1% penicillin, 1% streptomycin, and 1% glutamine (Gibco, Grand Island, NY, USA), was used to culture HUVECs at 37 °C in an environment containing 5% CO_2_. DMEM/F12 without FBS was used for synchronization. The NO donor SNP (100 µm) [19], the NOS inhibitor L‐NAME (500 µm), and the cysteine thiol‐reducing agent Tris(2‐carboxyethyl) phosphine hydrochloride (TCEP; 2 mm) were used to process cells.

### Dismutase activity assay

The dismutase activity was detected with a total SOD assay kit (Solarbio, Beijing, China) by colorimetric assay. The dismutase enzyme activity with an inhibitory rate of 50% in the xanthine oxidative coupling reaction system is defined as the enzyme activity unit [20].

### Measurement of the intracellular NO content

Diaminofluorescein‐FM diacetate (DAF‐FM DA Cat: S0019; Beyotime, Shanghai, China) was used to determine the NO content in HUVECs [21]. Treated cells were loaded with DAF‐FM DA (5 µm) at 37 °C for 20 min. An excitation wavelength of 495 nm and an emission wavelength of 515 nm were used to observe the fluorescence with the confocal laser scanning microscope (Olympus, Tokyo, Japan).

### Terminal deoxynucleotidyl transferase‐mediated dUTP nick‐end labeling (TUNEL) assay

The *in situ* apoptosis detection kit (Roche, Basel, Switzerland) was used to detect endothelial cell apoptosis [22]. After discarding the medium, cells were fixed with 4% paraformaldehyde for 15 min. Then, permeabilization solution (0.125 g of BSA, 2.5 mL of PBS, and 7.5 μL of Triton X‐100) was used to incubate the cells at 37 °C for 30 min. The TUNEL reaction mixture was used to incubate cells in the darkness at 37 °C for 1 h and washed. Finally, the nuclei were stained by 4′,6‐diamidino‐2‐phenylindole‐containing antifluorescence quencher (Zhongshan Golden Bridge, Beijing, China). An excitation wavelength of 450–500 nm and an emission wavelength of 515–565 nm were used to observe the fluorescence of cells with the confocal laser scanning microscope. The nuclei of apoptotic cells exhibited green fluorescence.

### Dihydroethidine (DHE) staining

Treated HUVECs were stained with DHE (10 µm; Beyotime, Shanghai, China) at 37 °C in the darkness for 30 min and fixed with 4% paraformaldehyde [23]. The confocal laser scanning microscope was used to observe cells with an excitation wavelength of 535 nm and an emission wavelength of 610 nm.

### Bioinformatics analysis of S‐nitrosoproteome

Using S‐nitrosylation as the keyword to search the human S‐nitrosoproteomics literature in the PubMed, a total of 5 related references were selected, namely, Chen et al. identified 717 S‐nitrosylated proteins [24], Koo et al. [25] identified 84 S‐nitrosylated proteins, Zhang et al. identified 213 S‐nitrosylated proteins [26], Ben‐Lulu et al. identified 511 S‐nitrosylated proteins [27], and Mnatsakanyan et al. [28] identified 3632 S‐nitrosylated proteins. In the 5 articles 676 S‐nitrosylated proteins appeared more than 2 times. The enrichment analysis of the 676 proteins was performed by g:profiler (https://biit.cs.ut.ee/gprofiler). The apoptotic pathway was enriched in the KEGG database.

### Biotin switch assay for protein S‐nitrosylation detection

The HUVECs were divided into 3 groups: control, SNP, and SNP+TCEP. After treated with SNP or SNP+TCEP for 2 h, cells were washed with precooled PBS for 3 times, and 50 µL of Hens 1 solution (0.25 M Hepes, 100 µm neocuproine, 1 mm EDTA, 1‰ SDS, 1% Triton X‐100, 1% protease inhibitors, 1% phosphatase inhibitors, and 1% PMSF) were added. Then, cells were incubated on ice for 20 min, and 10 μL of the supernatant was retained as total protein. The remaining supernatant was incubated with MMTS (20 mm) for 20 min at 50 °C, and then, 900 μL of precooled acetone were added and incubated for 20 min at −20 °C. The mixture was centrifuged at 14,000 ***g*** for 10 min at 4 °C, and the supernatant was poured off. The precipitation was resuspended with 85 μL of Hens 1 solution, 5 μL of ascorbic acid (5 mm), and 10 μL of Biotin‐HPDP (1 mm) and incubated for 90 min at room temperature. Subsequently, 30 μL of NeutrAvidinTM was added into the mixture and incubated at 4 °C overnight. The beads were rinsed 3 times with Hens 2 solution (1 mm EDTA, 20 mm Hepes, 100 mm NaCl, and 0.5% Triton X‐100). Proteins eluted from beads were subjected to western blotting to detect protein S‐nitrosylation [29].

### Fluorescent probe assay for *in situ* detection of total S‐nitrosylated proteins

Total S‐nitrosylated protein level was detected by cellular protein S‐nitrosylation modification detection kit (Cayman, Ann Arbor, MI, USA) following the manufacturer's instructions. The S‐nitrosylated protein was observed under confocal microscopy with excitation wavelength of 490 nm and emission wavelength of 610 nm.

### SOD1 shRNA transfection

At 50% confluence, HUVECs were transfected with a scrambled shRNA or lentivirus‐delivered SOD1 shRNA (Cyagen Bioscience, Guangzhou, China) at the concentration of 7 × 10^5^ TU·mL^−1^ and supplemented with 5 mg·mL^−1^ polybrene. After 24 h of infection, the cells were cultured in fresh medium for another 48 h. HUVECs were treated with 3 μg·mL^−1^ puromycin for 2 weeks to screen the stable SOD1 knockdown cell line.

### Statistical analysis

The spss18.0 software (SPSS Inc., Chicago, IL, USA) was used for statistical analysis. Multiple groups were compared using one‐way analysis of variance followed by Bonferroni tests for data with equal variances or the Dunnett T3 test for data with unequal variances. *P* < 0.05 was statistically significant.

## Results

### NO inhibits cysteine‐dependent SOD1 monomerization

**Fig. 1:**
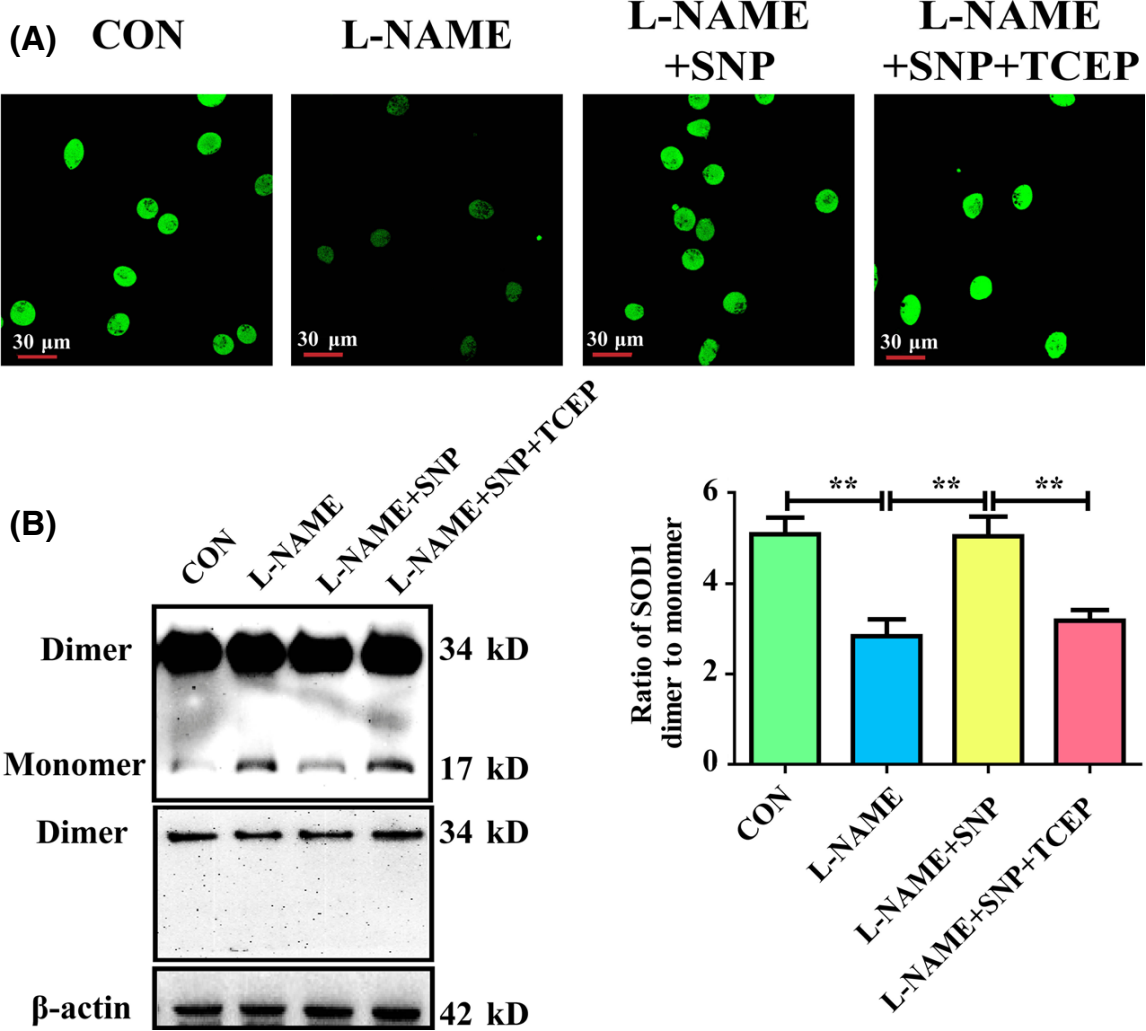
NO inhibits cysteine‐dependent SOD1 monomerization. (A) Fluorescence images of NO measured by DAF‐FM staining in L‐NAME‐treated HUVECs (*n* = 6). Scale bar = 30 μm. (B) Representative western blots of SOD1 in HUVECs treated with L‐NAME and quantitative analysis of the ratio of SOD1 dimer to monomer (*n* = 9). Data were expressed as the mean ± SD and were analyzed using one‐way ANOVA. **P < 0.01.

In order to reveal the effect of NO on SOD1 monomerization, HUVECs were treated with a NOS inhibitor L‐NAME to downregulate the endogenous eNOS/NO pathway. The results revealed that HUVECs treated with L‐NAME exhibited a decrease in NO content (Fig. 1A), and an increase in SOD1 monomer form (Fig. 1B) when compared with controls. While, SNP supplementation to upregulate the NO content reduced the level of SOD1 monomer form in L‐NAME‐treated HUVECs (Fig. 1A,B). These data suggested that NO inhibited the monomerization.

To explore whether NO inhibited SOD1 monomerization by acting on cysteine residues, L‐NAME‐treated HUVECs in the presence of SNP were incubated with TCEP, a specific reducer of cysteine thiol. The results showed that TCEP abolished the downregulation of SOD1 monomer level caused by SNP (Fig. 1B), demonstrating that NO inhibited cysteine‐dependent SOD1 monomerization.

### Cysteine‐dependent SOD1 monomer inhibition by NO upregulates dismutase activity and inhibits oxidative stress

**Fig. 2:**
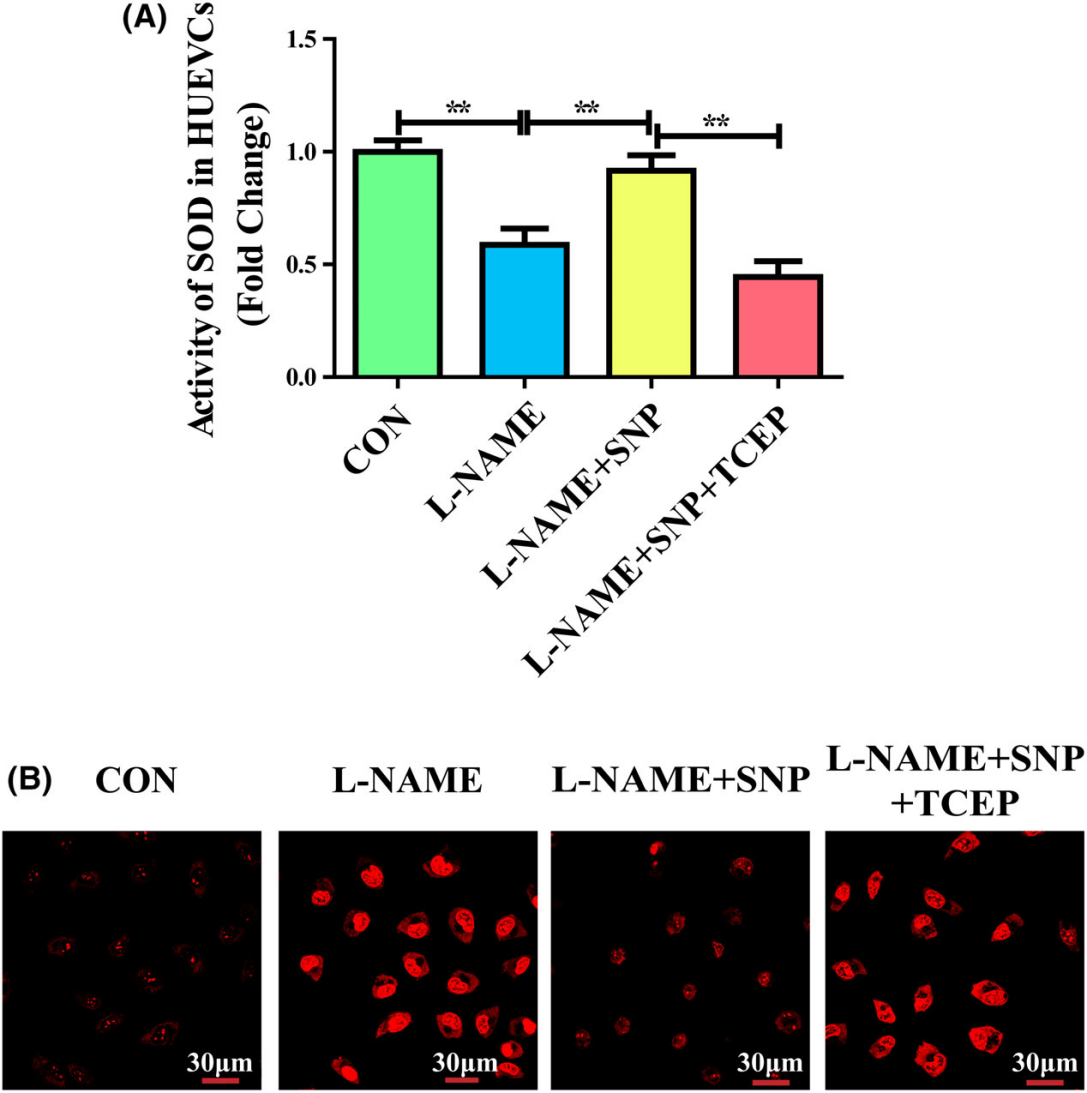
Cysteine‐dependent SOD1 monomer inhibition by NO upregulates dismutase activity and inhibits oxidative stress. (A) Quantification of dismutase activity in L‐NAME‐treated HUVECs (*n* = 6). Data were expressed as the mean ± SD and were analyzed using one‐way ANOVA. **P < 0.01. (B) Detection of superoxide in HUVECs treated with L‐NAME by DHE staining (*n* = 6). Scale bar = 30 μm.

We next investigated the potential effect of NO‐induced inhibition of cysteine‐dependent SOD1 monomerization on dismutase activity and oxidative stress. In the NOS activity inhibitor L‐NAME‐treated HUVECs, dismutase activity was significantly reduced, and ROS levels were increased when compared to those cells in control group. SNP treatment elevated the decreased dismutase activity and suppressed the increase in ROS, whereas TCEP abolished these effects of SNP (Fig. 2A,B). These results suggested that NO increased SOD1 activity and inhibited oxidative stress by inhibiting SOD1 monomerization.

### The inhibition of cysteine‐dependent SOD1 monomerization by NO inhibits endothelial cell apoptosis

**Fig. 3:**
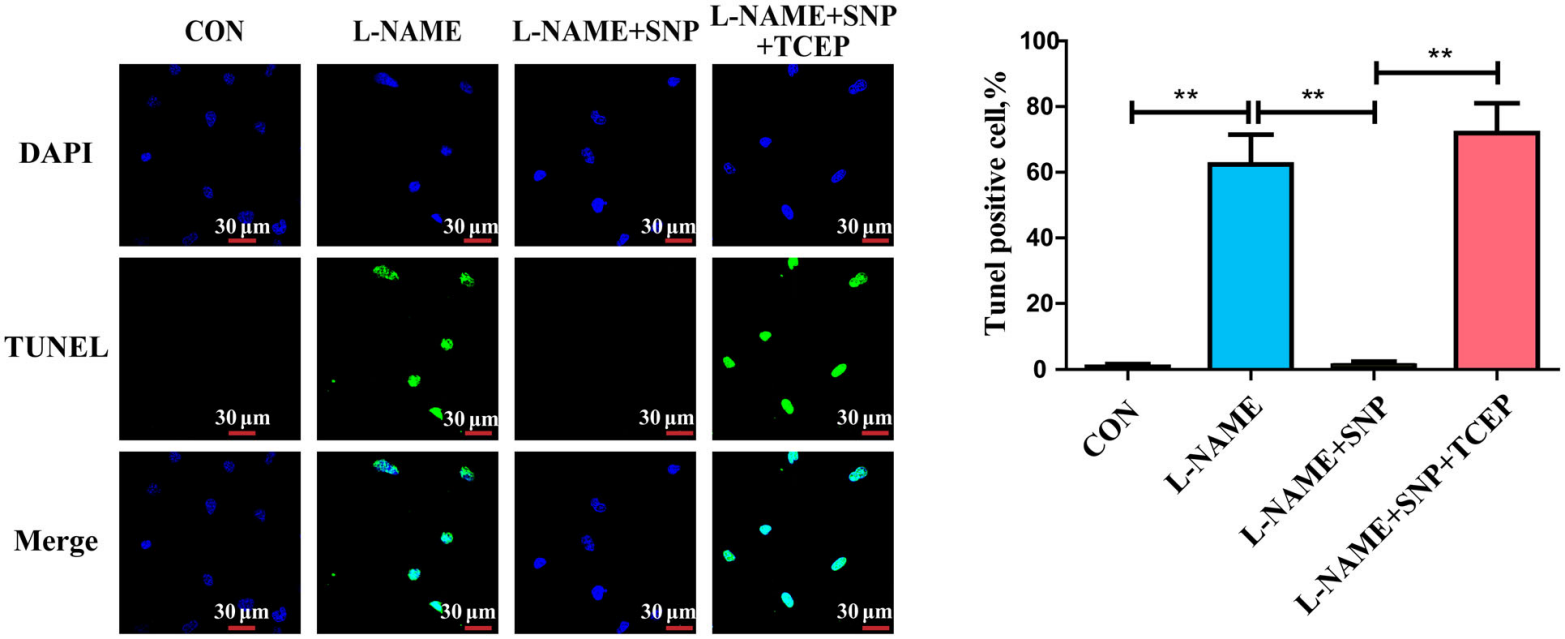
The inhibition of cysteine‐dependent SOD1 monomerization by NO inhibits endothelial cell apoptosis. TUNEL staining of apoptotic cells in HUVECs treated with L‐NAME (*n* = 9). Scale bar = 30 μm. Data were expressed as the mean ± SD and were analyzed using one‐way ANOVA. **P < 0.01.

To explore the potential function of cysteine‐dependent SOD1 monomer inhibition on SOD1 activation, we detected the apoptosis index in HUVECs. Compared with control group cells, the cell apoptosis index was increased in the NOS activity inhibitor L‐NAME‐treated cells (Fig. 3). The SNP exerted an anti‐apoptotic effect on HUVECs. Notably, SNP failed to protect L‐NAME‐treated HUVECs from apoptosis after TCEP treatment (Fig. 3). These data suggested that NO inhibited cysteine‐dependent SOD1 monomerization to protect against HUVEC apoptosis.

### NO S‐nitrosylated SOD1, and the apoptosis‐associated proteins caspase3 and TUBA4A

**Fig. 4:**
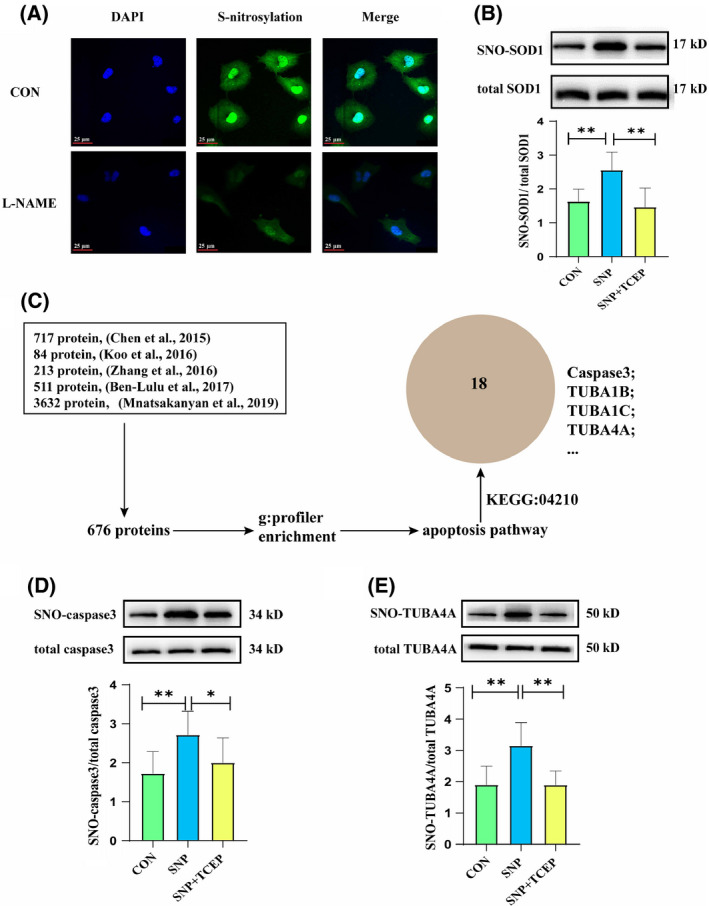
NO S‐nitrosylated SOD1, and the apoptosis‐associated proteins caspase3 and TUBA4A. (A) Total protein S‐nitrosylation in L‐NAME‐treated HUVECs (*n* = 6). Scale bar = 25 μm. (B) S‐nitrosylation of SOD1 in HUVECs treated with or without SNP or SNP plus TCEP (*n* = 10). Data were expressed as the mean ± SD and were analyzed using one‐way ANOVA. (C) Bioinformatics analysis of apoptotic proteins in human S‐nitrosoproteomics literatures. (D, E) S‐nitrosylation of caspase3 (D) and TUBA4A (e) in HUVECs treated with or without SNP or SNP plus TCEP (*n* = 10). Data were expressed as the mean ± SD and were analyzed using one‐way ANOVA. *P < 0.05, **P < 0.01.

To further explain why NO exerted these abovementioned effects, we evaluated the level of protein S‐nitrosylation in HUVECs. The results showed that S‐nitrosylated protein level in the L‐NAME‐treated HUVECs was decreased compared with the control group (Fig. 4A). Of note, SNP significantly promoted S‐nitrosylation of SOD1 protein, which was blocked by TCEP treatment in HUVECs (Fig. 4B). Furthermore, the bioinformatic study was conducted by analyzing 5 published human S‐nitrosoproteomics literatures (Fig. 4C). The result showed that there were 18 proteins in apoptosis pathway enriched by KEGG database, such as caspase3, TUBA1B, TUBA1C, and TUBA4A (Table 1). Therefore, we chose caspase3 and TUBA4A for further verification. Biotin switch assay showed that SNP facilitated S‐nitrosylation of caspase3 and TUBA4A, which was blocked by TCEP treatment in HUVECs (Fig. 4D,E). These results suggested that NO could induce S‐nitrosylation of SOD1, and the apoptosis‐associated proteins caspase3 and TUBA4A;, which might be involved in the anti‐apoptotic mechanism for NO.

**Table 1:** The apoptosis‐related proteins with S‐nitrosylation site.

ID	Gene name	Protein name	Reference
O14920	IKBKB	Inhibitor of nuclear factor kappa‐B kinase subunit beta	[24, 28]
P02545	LMNA	Prelamin‐A/C	[24, 26, 28]
P07339	CTSD	Cathepsin D	[27, 28]
P07384	CAPN1	Calpain‐1 catalytic subunit	[27, 28]
P07711	CTSL	Procathepsin L	[24, 28]
P07858	CTSB	Cathepsin B	[24, 27, 28]
P17655	CAPN2	Calpain‐2 catalytic subunit	[24, 27, 28]
P19838	NFKB1	Nuclear factor NF‐kappa‐B p105 subunit	[24, 28]
P42574	CASP3	Caspase‐3	[24, 28]
P60709	ACTB	Actin, cytoplasmic 1	[24, 25, 27]
P63261	ACTG1	Actin, cytoplasmic 2	[25, 27]
P68363	TUBA1B	Tubulin alpha‐1B chain	[24, 27]
P68366	TUBA4A	Tubulin alpha‐4A chain	[24, 28]
P98170	XIAP	E3 ubiquitin‐protein ligase XIAP	[24, 27, 28]
Q04206	RELA	Transcription factor p65	[24, 28]
Q99683	MAP3K5	Mitogen‐activated protein kinase kinase kinase 5	[24, 27]
Q9BQE3	TUBA1C	Tubulin alpha‐1C chain	[27, 28]
Q9UKK3	PARP4	Protein mono‐ADP‐ribosyltransferase PARP4	[27, 28]

### SOD1 is required for the inhibitory effect of NO on endothelial cell apoptosis

**Fig. 5:**
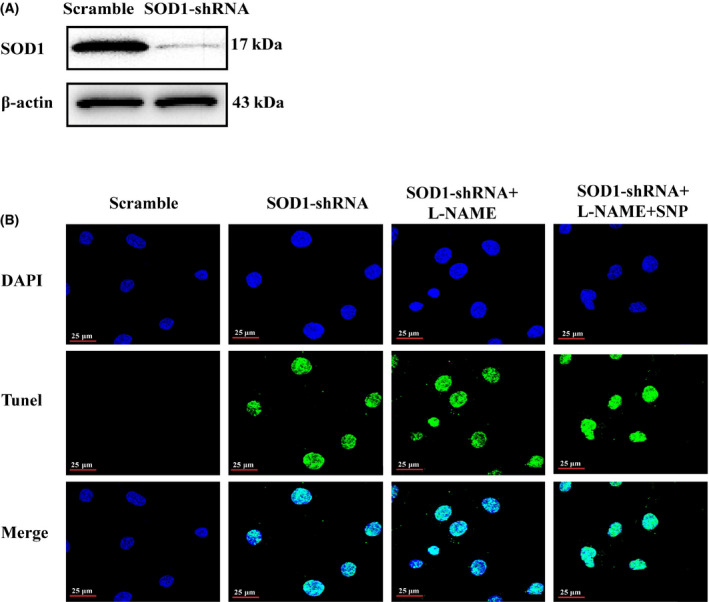
SOD1 is required for the inhibitory effect of NO on endothelial cell apoptosis. (A) Representative western blots of SOD1 in HUVECs transfected with scramble or SOD1 shRNA (*n* = 3). (B) TUNEL staining of apoptotic cells in HUVECs treated with transfected with scramble or SOD1 shRNA in the presence or absence of L‐NAME or SNP (*n* = 9). Scale bar = 25 μm.

To further confirm the importance of SOD1 in the regulatory role of NO in endothelial cell apoptosis, we knocked down SOD1 by shRNA in HUVECs. Compared with scramble group, SOD1 shRNA transfection reduced SOD1 protein expression (Fig. 5A) and induced apoptosis in HUVECs (Fig. 5B). Of note, SNP treatment could no longer protect SOD1 knocked down endothelial cells against apoptosis (Fig. 5B). These results confirmed that SOD1 was required for the inhibitory effect of NO on endothelial cell apoptosis.

## Discussion

In this study, we firstly demonstrated that NO inhibited cysteine‐dependent SOD1 monomerization to promote dismutase activity and inhibit oxidative stress, thereby protecting endothelial cells from apoptosis. Pharmacologic inhibition of endogenous NO content resulted in the increase of monomers, leading to the decreased dismutase activity, enhanced oxidative stress and eventually endothelial cell apoptosis. Thus, our findings revealed that the inhibition of cysteine‐dependent SOD1 monomerization was a novel mechanism by which NO inhibited endothelial cell apoptosis.

Excessive apoptosis of endothelial cells severely interferes with endothelial integrity and endothelial function, resulting in endothelial damage, which is closely associated with the occurrence and the development of various cardiovascular diseases [2, 3, 4, 5, 6]. NO, which is predominantly synthesized by eNOS in endothelial cells, is a vasoactive substance secreted by endothelial cells [7]. NO has many beneficial effects, such as relaxing blood vessels, preventing platelet aggregation, inhibiting leukocyte adhesion, and controlling the proliferation of vascular smooth muscle cells [30, 31, 32, 33]. NO displays pro‐apoptotic or anti‐apoptotic effects [34] depending on its effective concentration, cell types, and microenvironment. High concentration of NO produced by iNOS induced apoptosis in macrophages [35]. Excessive extracellular superoxide anion derived from neutrophils under inflammatory conditions could react with NO to form high level of peroxynitrite, which led to a rapid apoptosis of neutrophils [36]. In contrast, low concentration of NO and peroxynitrite was reported to inhibit apoptosis in endothelial cells [37] and hepatocytes [38]. The physiological concentration of NO produced by eNOS has anti‐apoptotic and cytoprotective effects [39, 40]. Andrographolide, hydrogen sulfide, and oxymatrine were reported to inhibit endothelial cell apoptosis through upregulating Akt/eNOS pathway [41, 42, 43]. Moreover, eNOS deficiency increased endothelial cell apoptosis and aggravated renal injury in mice with remnant kidney [44]. In our study, the treatment with NOS inhibitor L‐NAME markedly induced apoptosis in HUVECs, whereas NO supplementation protected HUVECs against apoptosis, suggesting that eNOS/NO had protective effect against endothelial cell apoptosis.

Nitric oxide affects cell apoptosis through diverse signaling pathways, which increases the complexity of NO action. This stimulated us to explore the unknown mechanism underlying NO signaling. In spinal cord motor neurons, SOD1 blocks iNOS‐mediated apoptosis [45]. Furthermore, NO upregulates SOD1 expression in vascular smooth muscle cells to inhibit neointimal hyperplasia [46]. These findings imply that SOD1 might be a potential target of NO signaling. SOD1 activity is not only determined by protein expression but also regulated by post‐translational modifications. The homodimer is the most stable existence and active form of SOD1. The dissociation of SOD1 dimer into monomers which were more likely to misfold and aggregate could inhibit SOD1 activity [47]. NO can induce post‐translational modification of specific cysteines to regulate the biological activity of target proteins. However, little has been known about the post‐translational modification of SOD1 by NO. In the present study, we found that NO inhibited SOD1 monomerization and promoted dismutase activity in HUVECs. Suppression of the eNOS/NO pathway in HUVECs resulted in the increase of SOD1 monomers, downregulated dismutase activity, and increased ROS accumulation, whereas NO supplementation rescued these effects. These findings demonstrated that NO inhibited SOD1 monomerization to promote SOD1 activity.

The SOD1 homodimer structure is stabilized by an intrasubunit disulfide bond between Cys57 and Cys146. The cleavage of the intramolecular disulfide can predispose the SOD1 dimer to dissociate and downregulate SOD1 activity [48]. Among the other two free cysteines, Cys6 is located inside the SOD1 structure, which is not easily accessible for reaction. Due to the strong activity of Cys111 and the proximity of Cys111 residues in adjacent SOD1 monomers, the thiol‐disulfide bond exchange between these two adjacent cysteines restores the monomer–dimer equilibrium [49]. NO modifies the thiol group on the cysteine residues of some enzymes (such as matrix metalloproteases) by disulfide bond formation and other modifications, thus increasing the enzyme activity [50, 51]. Based on the above findings, we speculated that NO inhibited SOD1 monomerization by acting on the cysteine residues of SOD1. To prove this hypothesis, we used TCEP, the specific reducing agent of cysteine thiols. TCEP successfully abolished NO‐induced inhibition of SOD1 monomerization in HUVECs, indicating that NO inhibited monomeric SOD1 by acting on cysteine thiol. Furthermore, TCEP eliminated NO‐induced dismutase activity and its inhibitory effect on ROS and endothelial cell apoptosis. These data suggest that the inhibition of cysteine‐dependent SOD1 monomerization mediates the protective effect of NO on endothelial cells, including the activation of SOD1 to antagonize oxidative stress and apoptosis.

NO can S‐nitrosylate cysteine to form protein S‐nitrosothiols, thereby regulating protein structure and function. Previous study showed that the S‐nitrosylation of β‐arrestin1/2 by NO mediates homodimerization [52]. However, it is still unclear whether NO S‐nitrosylates SOD1 protein. Our study showed that SNP could promote SOD1 S‐nitrosylation, while TCEP blocked this effect, indicating that S‐nitrosylation of sulfhydryl group at SOD1 cysteine by NO might be related to the inhibition of SOD1 monomerization. Schonhoff et al [53] showed that misfolded SOD1 mutants caused S‐nitrosothiol depletion, disrupting the function and/or subcellular localization of proteins regulated by S‐nitrosylation, among which some were related to the apoptosis induction. In the present study, we found that S‐nitrosylated protein level in the L‐NAME‐treated HUVECs was decreased, while supplementation of SNP increased it. Moreover, SNP facilitated the S‐nitrosylation of caspase3 and TUBA4A that were enriched in the apoptosis pathway by the KEGG database based on a collective human S‐nitrosoproteome. Furthermore, we knocked down SOD1 in HUVECs and found that SNP could no longer protect cells against apoptosis, suggesting that SOD1 is required for the inhibitory effect of NO on endothelial cell apoptosis.

In summary, our data showed a novel mechanism by which NO protected the endothelial cell against oxidative stress‐induced apoptosis and found that NO inhibited cysteine‐dependent SOD1 monomerization and thereby blocked the inactivation of SOD1. Considering the pivot role of SOD1 in the balance of anti‐/pro‐oxidative system, the inactivation of SOD1 is considered to be an important pathogenesis of many diseases such as cardiovascular disease, aging, and cancer. Therefore, the present study might elucidate a new therapeutic principle behind NO in the treatment of oxidative stress‐related diseases and promote the design and the clinical application of NO‐related drug.

## Conflict of interest

There is no conflict of interest in this manuscript.

## Author contributions

HP, SZ, ZZ, XW, YH, and HJ conceived and designed the project. HP, SZ, ZZ, and XW performed the experiments. HP, SZ, ZZ, XT, and LZ analyzed and interpreted the data. HP, ZZ, YH, HJ, and JD wrote the article. All authors have read and approved the final manuscript.

## Data Availability

The data are available by contact with the corresponding authors.
